# Us for us: reflections of a journey by people who use drugs in East Africa to shape HIV, hepatitis and other relevant health policies

**DOI:** 10.1002/jia2.25730

**Published:** 2021-06-30

**Authors:** Aggrey A Odhiambo

**Affiliations:** ^1^ Health and Rights Program Open Society Initiative for Eastern Africa Nairobi Kenya

**Keywords:** drug use, agency, transformative engagement, persons who use drugs, East Africa, policies, advocacy

## 1 Targeted financial and capacity support help PWID lead and improve HIV responses in their communities in East Africa

HIV prevalence, incidence and risk among people who inject drugs (PWID) are far greater than among overall populations, even in generalized epidemics, such as those in most of sub‐Saharan Africa. Some successes in overcoming the huge challenges to addressing these disparities have occurred in East Africa in recent years. These have been driven largely by deliberate efforts of members of drug‐using communities themselves with the support of development and financing partners, including the Open Society Foundations (OSF), the United Nations Office on Drugs and Crime (UNODC), Médecins du Monde, Aidsfonds and the Global Fund to Fight AIDS, Tuberculosis and Malaria [1, 2]. Examples from three countries in this commentary highlight some of the important types and impacts of activities supported by OSF, an active player in supporting the health and human rights of PWID around the world.

## 2 Kenya: real progress recorded

The strengthened involvement of PWID in HIV programming in Kenya can be traced back to 2008, when the OSF Public Health Program initiated catalytic support to networks and groups of people who use drugs and harm reduction organizations. The main data points at the time included an estimated HIV prevalence of 49.5% among PWID, based on a 2006 study of a cohort in Mombasa [3] and a population size estimate of 16,003 PWID in the country by a 2008 Kenya Modes of Transmission (KMOT) study [4].

Since that time, OSF has supported 11 organizations to implement harm reduction work in Kenya. Nine were non‐governmental organizations (NGOs) employing peer‐led approaches to enhance access to harm reduction services and support access to justice for people who use drugs, including through differentiated approaches to ensure gender equity; and two were government agencies working on drug control and HIV prevention [5, 6]. Other key areas of work funded by OSF in which people who use drugs have had leadership roles [1] include targeted economic empowerment for the community and training and awareness‐raising initiatives among law enforcement staff as a way to better safeguard the safety and security of people who use drugs [7, 8, 9].

Recent data indicate progress since OSF became involved in reducing the severity of HIV and other debilitating health outcomes common among PWID related to injecting behaviour. According to a 2017 report, HIV prevalence among PWID in Kenya was 18.3%, nearly three times lower than what was estimated ten years earlier [10]. Hepatitis C virus (HCV) prevalence among PWID is currently estimated at 18% (NASCOP, 2020), a decline from 22.2% reported in 2008 when a study on a cohort of people who use drugs established HCV prevalence compared to the general population with a prevalence of 0.2‐0.9% [11].

## 3 Tanzania: harm reduction recognized

In 2006, HIV prevalence among PWID in Tanzania stood at around 25% compared with an estimated 5.7% in the general population [12]. Tanzania had an estimated 30,000 PWID, and HIV prevalence among the population was estimated to be 35% [13]. HCV prevalence among PWID in Dar es Salaam ranged from 44% to 57% in 2018 [14, 15]. These findings attest to the existence of a major ongoing health concern in the PWID community. Other major challenges for the efforts in Tanzania to date include, in recent years, limitations on access to services due to state‐sanctioned restrictions on targeted programming for key populations [16].

Since 2009, OSF has supported six civil society organizations and two government agencies to provide harm reduction services in Tanzania, with a focus on involving and building the capacity of people who use drugs. Components of this support have included legal empowerment, advocacy for legal and social reforms, community education, economic empowerment for people who use drugs and gendered harm reduction targeting women who use drugs.

The enhanced capacity of PWID has created momentum that helped ensure that harm reduction is recognized in the national HIV response with gender as a factor in the design [1]. Consequently, the number of PWID accessing harm reduction services has continued to increase, with an estimated 7,600 people currently enrolled in medication‐assisted treatment (MAT) provided through eight sites [3].

## 4 Uganda: gaining access to policy spaces

In Uganda, there are limited data for key populations. However, from available studies, HIV is concentrated in key and priority populations. In 2017, HIV prevalence among PWID was estimated at between 11% and 34% [17]. Estimates from the most recent national size estimate for PWID indicate an upper bound of 11,034 [17], with HIV prevalence among PWID estimated at 17‐20% against a national prevalence of 6.2%, according to the Uganda AIDS Commission in 2019. HCV prevalence among people who use drugs in Uganda was recently estimated to be 1.6% [19, 20]. Since 2016, OSF has supported three NGOs to undertake work on addressing barriers to harm reduction services in Uganda, including access to justice [18], addressing stigma and social exclusion, gender‐targeted interventions for people who use drugs, and building the movement for the enhanced voice and agency of the community.

The support has seen successful advocacy engagements with government stakeholders, including law enforcement, media and policy makers, and opinion leaders from Uganda’s Ministry of Health and the Ministry of Internal Affairs. These efforts have enabled organizations led by people who use drugs to gain access to policy spaces, such as national MAT taskforce meetings and Global Fund country coordinating mechanisms. Together, these engagements have given the Uganda Harm Reduction Network (UHRN) and other harm reduction stakeholders more influence and convening power that they continue to capitalize on for the benefit of people who use drugs.

## 5 Conclusions

OSF and other funders supporting people who use drugs have enabled the nurturing of sustainable home‐grown solutions to address the systemic challenges that pose hurdles for PWID’s quest for health rights. Moving forward, similar support for community capacity‐building and leadership to develop and sustain harm reduction services is likely to be even more important in the region. More broadly, it is crucial in the face of a shrinking space for civil society in general, financing and health systems constraints due to COVID‐19, and minimal progress in addressing critical structural barriers, such as criminalization of possession and use of drugs.

## COMPETING INTERESTS

The authors have declared no conflict of interest.

## ABBREVIATIONS

CSO, civil society organization; HCV, hepatitis C virus; KANCO, Kenya AIDS NGO Consortium; MAT, medication‐assisted treatment; NGO, non‐governmental organization; OSF, Open Society Foundations; PWID, people who inject drugs.
